# Influence of Maternal Habitat on Salinity Tolerance of *Zygophyllum coccineum* with Regard to Seed Germination and Growth Parameters

**DOI:** 10.3390/plants9111504

**Published:** 2020-11-06

**Authors:** Elsayed Mohamed, Ahmed M. M. A. Kasem, Adil A. Gobouri, Amr Elkelish, Ehab Azab

**Affiliations:** 1 Botany & Microbiology Department, Faculty of Science (Assiut), Al-Azhar University, Assiut 71524, Egypt; amkasem@azhar.edu.eg; 2 Department of Chemistry, College of Science, Taif University, P.O. Box 11099, Taif 21944, Saudi Arabia; a.gobouri@tu.edu.sa; 3 Botany Department, Faculty of Science, Suez Canal University Ismailia, Ismailia 41522, Egypt; amr.elkelish@science.suez.edu.eg; 4 Department of Biotechnology, College of Science, Taif University, P.O. Box 11099, Taif 21944, Saudi Arabia; e.azab@tu.edu.sa; 5 Botany and Microbiology Department, Faculty of Science, Zagazig University, Zagazig, 44519, Egypt

**Keywords:** seed germination, halophytes, maternal habitats, *Zygophyllum coccineum*, superoxide dismutase, esterase

## Abstract

*Zygophyllum coccineum* is a facultative halophyte widely distributed in desert wadis and coastal areas in Egypt. Here, we investigated the influences of maternal habitat on tolerance to salt stress during germination and seedling growth under salinity (0, 100, 200, 400 mM NaCl) of three populations of *Z. coccineum* from a saline habitat (Manzala coast) and non-saline habitats (Wadi Houf and Wadi Asyuti). In all populations, seed germination started within two days in distilled water but germination indices were reduced significantly with salt level increase. Germination percentage was not significantly greater for seeds from non-saline habitats than for those from the saline habitat under moderate salinity (100, 200 mM NaCl), but only seeds from the saline habitat were able to germinate under high salt stress (400 mM NaCl). Germination recovery was greater for seeds from the saline habitat compared to non-saline populations. At the seedling level, the Manzala population showed the lowest inhibition of shoot length and leaf area under salinity (200 and 400 mM NaCl) compared to non-saline habitats. In the same context, the Manzala population had the maximum chlorophyll a content, superoxide dismutase and esterase activities under salinity compared to non-saline populations, but salinity had a non-significant effect on chlorophyll b between the three populations. Carotenoids were enhanced with the increase of salt levels in all populations. These results suggest the salt tolerance of Manzala population is derived from maternal salinity and adaptive plasticity of this species may play an important role in the wide distribution of *Z. coccineum*.

## 1. Introduction

Wide ecological amplitude species have two unique mechanisms to overcome the impacts which arise from environmental changes: (1) local adaptation and (2) phenotypic plasticity [1]. Local adaptation emerges due to the selection of local habitat especially in the presence of low gene flow. This situation leads to favorable traits that enhance the fitness of the population in the local habitat [2]. Therefore, local adaptation plays a fundamental role in evolution, conservation and coping mechanisms towards global climate change [3]. Phenotypic plasticity is the ability of a single genotype to produce different phenotypes according to changes in the environment [4]. This mechanism is non-adaptive genetic differentiation and occurs faster than local adaptation [1,5].

Climate changes increase soil salinity and drought, both stresses cause losses in agricultural production and affect the distribution of wild plants globally, for example, plant species become widespread in newly favorable habitats and decrease in unfavorable areas [6,7,8]. However, species with phenotypic plasticity can survive under these new drastic conditions [9]. Maternal effects are the influences of edaphic and ecological factors during the life cycle of mother plants on behavior of offspring through transgenerational defense initiation [10,11]. Maternal environmental effects are considered as a type of epigenetic modification or intergenerational phenotypic plasticity, and thus maternal habitats affect the behavior of offspring [12]. In contrast, maternal environmental effects may induced adaptive plasticity through offspring, for instance, *Plantago lanceolate*, a perennial herb which survive in mowed and unmowed areas, showed cumulative fitness when offspring were grown in the same maternal environments [13]. Galloway et al. [14] reported the adaptive maternal environmental effects on *Campanula americana*; maternal light effects promoted the fitness of offspring due to increasing germination and influencing the season of germination when grown in their maternal habitat.

One of the most popular kinds of maternal habitat effects derived from maternal salinity, regarded germination behavior, seed dormancy, and seedling establishment, was in response to maternal salinity effects in many plant species, as observed in *Dipascus fullonum* subsp. *sylvestris*, *Ambrosia artemisiifolia* and *Suaeda vermiculata* [15,16,17]. Salinity induces inhibition of seed germination percentage and plant growth due to the prevention of water uptake, harmful effects of toxic ions, or both [18,19]. Furthermore, salinity leads to oxidative stress due to enhancing the generation of reactive oxygen species such as hydrogen peroxide, superoxide and hydroxyl radicals [20]. Oxidative stress causes a reduction in photosynthetic pigments and plant productivity [21]. Chlorophyll content was used as criteria for discrimination between salt-sensitive and salt tolerance varieties of many plants such as *Gossypium hirsutum*, *Triticum aestivum* and *Medicago sativa* [22,23,24].

Plants alleviate oxidative damage under saline conditions due to non-enzymatic and enzymatic efficient antioxidant mechanisms. Superoxide dismutase (SOD), catalase (CAT) and ascorbate peroxidase (APX) are the most common antioxidant enzymes [25,26,27]. Superoxide dismutase plays a vital role in the dismutation of superoxide anions to molecular oxygen and hydrogen peroxide [28]. Furthermore, esterase activity is implicated in plant development regulation, defense response and is considered as a biomarker for salt tolerance in plants [29,30].

*Zygophyllum* are annual or perennial shrubs; it includes 80 species, widely distributed in the Mediterranean region, Australia and Asia in desert and saline areas. This genus is represented in Egypt by *Z. simplex, Z. album*, *Z. aegyptium*, *Z. coccineum*, *Z. decumbens*, *Z. dumosum* and *Z. fabago* [31]. *Z. coccineum* is a perennial herb with many erect branched stems and its leaves are 2-foliate, glabrous and bright green. This plant can be propagated by seeds and cuttings, the cuttings should be taken and cultivated in late March in nurseries and then transferred to natural fields in autumn [31,32]. *Z. coccineum* has medicinal value as an antidiabetic and antioxidant, and ecological value as a bio-accumulator for heavy metals from the soil and water [33,34,35,36]. Due to unpalatability and the arid conditions of Egypt, it is expected to have low dispersal for its seeds. Surprisingly, this species has the largest distribution in Egypt compared to other *Zygophyllum* species; it invades various habitats and different soil types [37]. We hypothesized that maternal environmental effects, especially maternal salinity, contribute to the widespread distribution of *Z. coccineum*. The present work aims to evaluate the effect of maternal salinity on seed germination and growth of *Z. coccineum* from saline (Manzala coast) and desert habitats (Wadi Houf and Wadi Asyuti) through the study of the influence of salinity on seed germination, recovery, growth parameters, chlorophyll contents, superoxide dismutase and esterase activities of the studied populations.

## 2. Results

### 2.1. Seed Mass and Length

**Figure 1:**
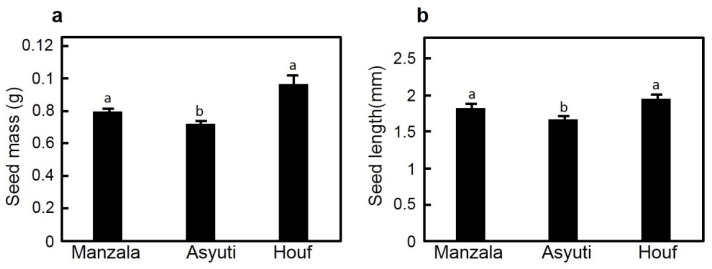
Comparison of (**a**) seed mass and (**b**) seed length of *Zygophyllum coccineum* from Manzala coast, Wadi Asyuti and Wadi Houf. Value represents the mean of 100 seeds ± standard error, *p*-value < 0.05. For variables denoted with the same letter, the difference is not statistically significant.

Mass of seeds from Manzala (0.08 g) or Wadi Houf (0.097 g) were significantly heavier compared to the Wadi Asyuti specimen (0.072 g). In the same context, seed length also showed a significant increase for Manzala and Wadi Houf populations compared to those from Wadi Asyuti (Figure 1).

### 2.2. Effect of Salinity on Germination

**Figure 2:**
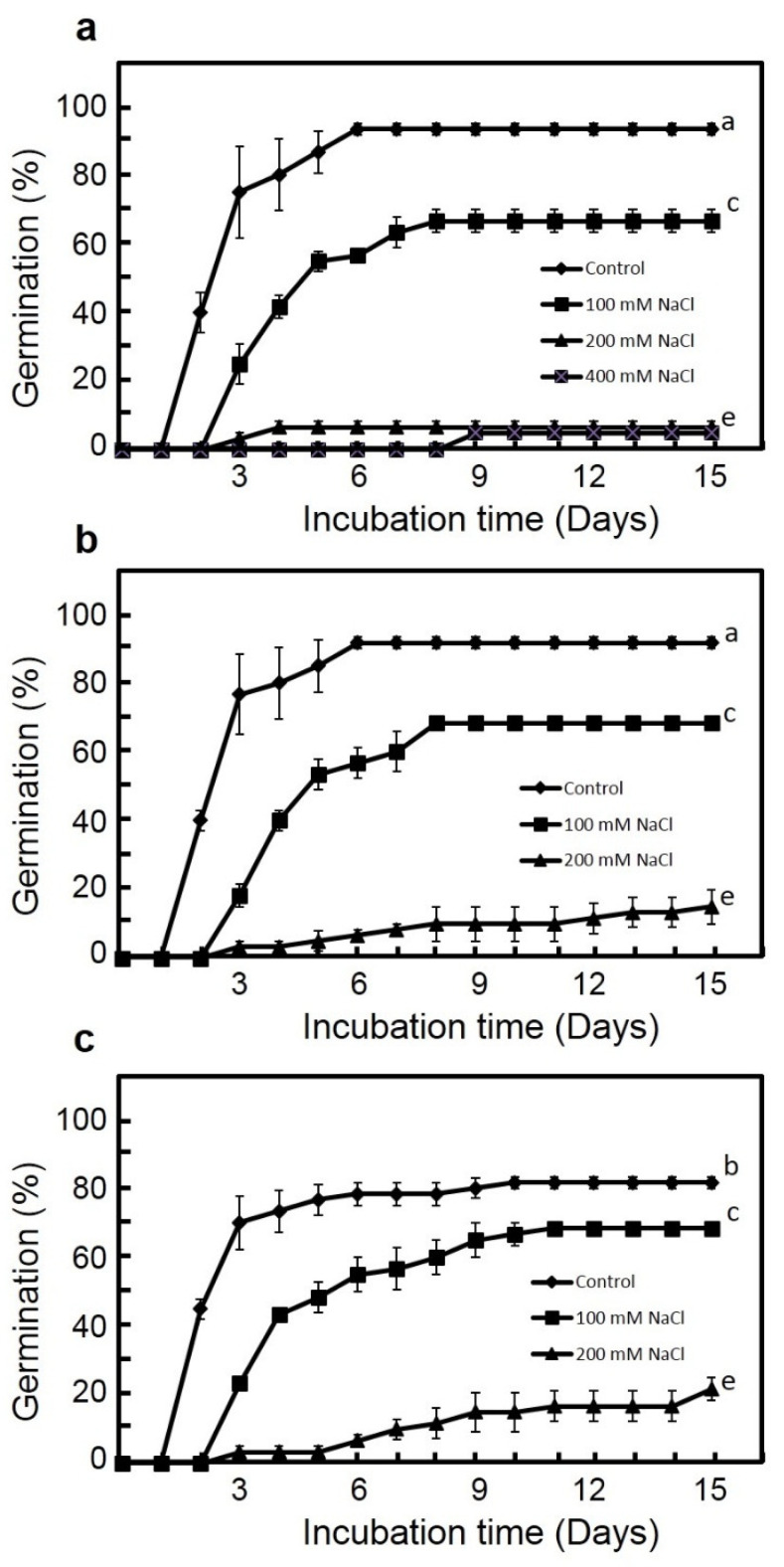
Germination of *Zygophyllum coccineum* seeds from (**a**) Manzala coast, (**b**) Wadi Asyuti and (**c**) Wadi Houf in distilled water, 100, 200 and 400 mM NaCl for 15 days. Value represents 3 replicates ± standard error. For variables denoted with the same letter, the difference is not statistically significant.

**Figure 3:**
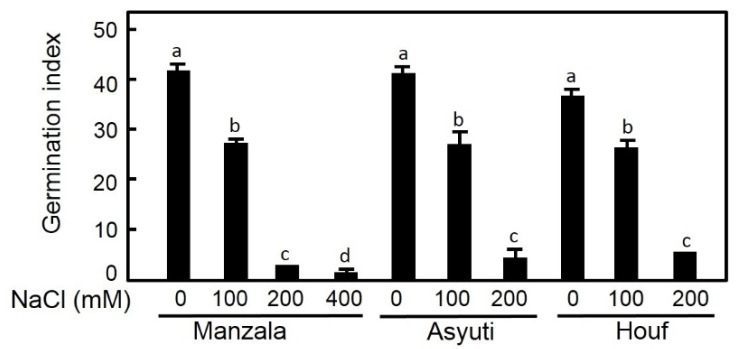
Germination rate index of *Zygophyllum coccineum* seeds from Manzala coast, Wadi Asyuti and Wadi Houf in distilled water, 100, 200 and 400 mM NaCl for 15 days. Value represents the mean of 3 replicates ± standard error. For variables denoted with the same letter, the difference is not statistically significant.

Two-way ANOVA showed significant effects of habitat and salinity on both germination and recovery percentages (Table 1). The maximum germination percentage was recorded in distilled water for the Manzala population (93.3%) followed by Wadi Asyuti (91.6%) and then Wadi Houf populations (81.6%). Seeds from Wadi Asyuti and Wadi Houf populations (non-saline areas) showed higher germination percentages compared to the Manzala population (saline habitat) under low and moderate salinity (100 and 200 mM NaCl). Interestingly, germination under high salinity (400 Mm NaCl) was only observed in Manzala seeds while it was completely inhibited in Wadi Asyuti and Wadi Houf populations under the same salinity (Figure 2). Moreover, salinity significantly influenced germination indices; the three populations showed very rapid germination in distilled water and the germination rate indices (GRI) decreased with the increase of salt level. However, GRI did not significantly differ among populations in all salinity levels (Figure 3).

**Figure 4:**
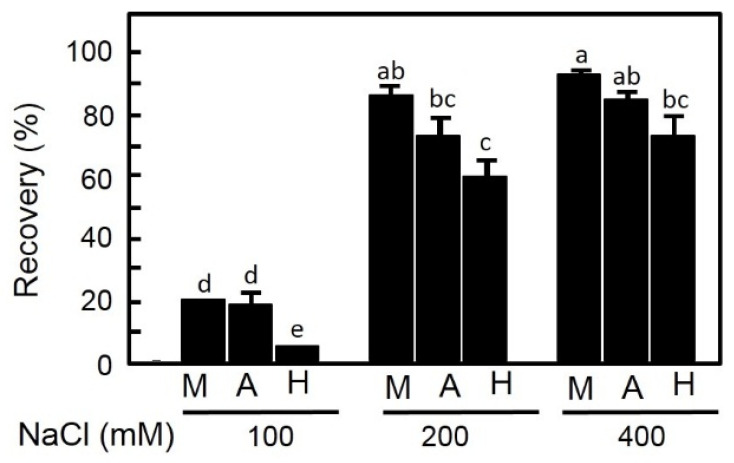
Germination percentages of *Zygophyllum coccineum* seeds recovered from 100, 200 and 400 mM NaCl for 15 days. Value represents the mean of 3 replicates ± standard error. For variables denoted with the same letter, the difference is not statistically significant.

For the recovery percentage, it was increased according to the previous salt treatment for all populations. For lower salinity treatment (100 mM NaCl), recovery of Wadi Houf seeds was significantly lower compared with Manzala and Wadi Asyuti seeds. In contrast, the maximum germination recovery from moderate and high saline concentrations (200 and 400 Mm NaCl) was observed in seeds from Manzala compared to Wadi Asyuti and Wadi Houf seeds (Figure 4).

### 2.3. Growth Parameters

**Figure 5:**
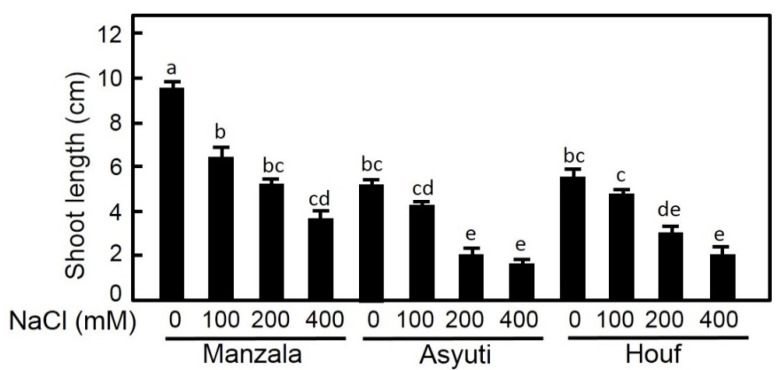
Influences of different salt concentrations (0, 100, 200 and 400 mM NaCl) on shoot length in *Z. coccineum* for 45 days. Value represents the mean of 5 replicates ± standard error, *p*-value < 0.05. For variables denoted with the same letter, the difference is not statistically significant.

**Figure 6:**
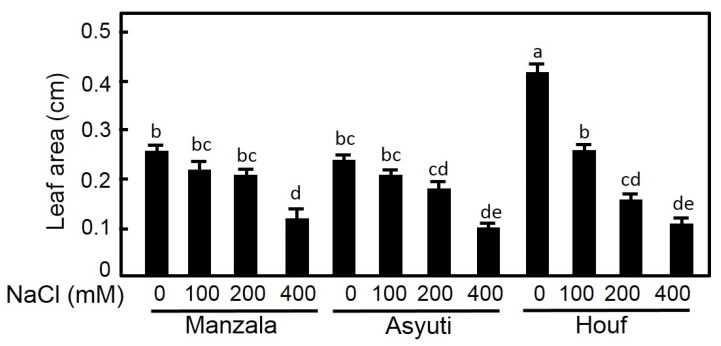
The effects of different salt concentrations (0, 100, 200 and 400 mM NaCl) on leaf area in *Z. coccineum* for 45 days. Value represents the mean of 5 replicates ± standard error, *p*-value < 0.05. For variables denoted with the same letter, the difference is not statistically significant.

Results of two-way ANOVA showed that habitat, salinity and their interaction had significant effects on shoot length and leaf area (Table 1). Salinity treatments inhibited shoot length and leaf area of all populations (Figure 5). The reduction of shoot length was significant under all salinity levels (100, 200 and 400 mM NaCl) and shoot length was higher in the Manzala population than in Wadi Asyuti and Wadi Houf seedlings in both non-saline and saline conditions. In contrast, leaf area of the Manzala population showed a significant reduction in 400 mM NaCl, in 200 and 400 mM NaCl for the Wadi Asyuti population, and under all salinity treatments for the Wadi Houf population which had the maximum leaf area in the absence of salinity (Figure 6).

### 2.4. Photosynthetic Pigments

**Figure 7:**
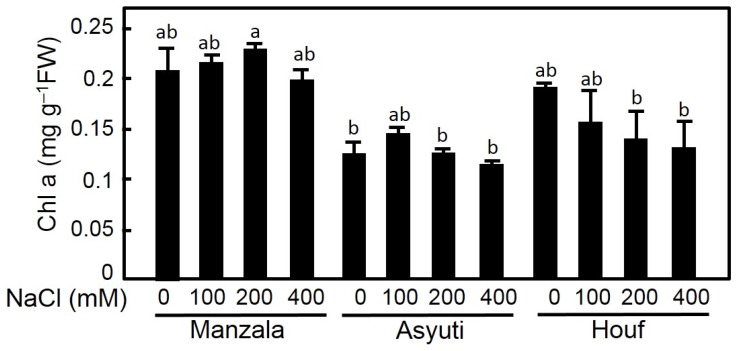
Effects of 45 days salt treatments (0, 100,200 and 400 mM NaCl) on chlorophyll a in *Z. coccineum* from Manzala, Wadi Asyuti and Wadi Houf. Value represents the mean of 3 replicates ± standard error, *p*-value <0.05. For variables denoted with the same letter, the difference is not statistically significant.

**Figure 8:**
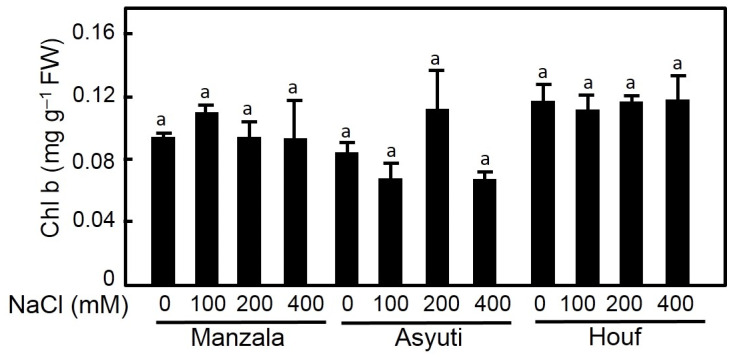
Effects of 45 days salt treatments (0, 100, 200 and 400 mM NaCl) on chlorophyll b in *Z. coccineum* from Manzala, Wadi Asyuti and Wadi Houf. Value represents the mean of 3 replicates ± standard error, *p*-value < 0.05. For variables denoted with the same letter, the difference is not statistically significant.

**Figure 9:**
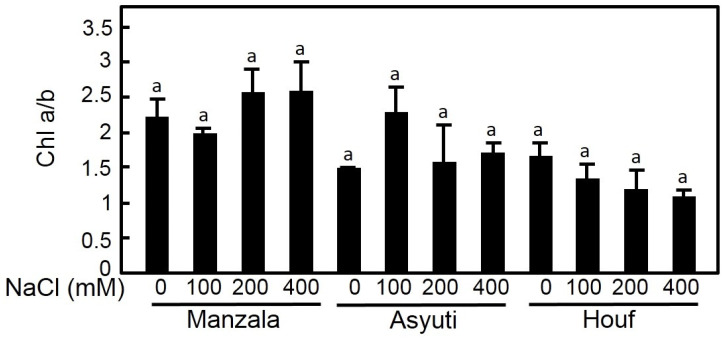
The effects of different salt concentrations (0, 100, 200 and 400 mM NaCl) on Chl a/b in *Z. coccineum* for 45 days. Value represents the mean of 5 replicates ± standard error, *p*-value < 0.05. For variables denoted with the same letter, the difference is not statistically significant.

Maternal habitat had a significant effect on both chlorophyll a and b contents. Chl a was promoted by 100 and 200 mM NaCl treatments in Wadi Asyuti and Manzala populations and it was decreased in response to salinity in Wadi Houf plants (Figure 7). Regarding habitat, maximum Chl a content was recorded in the Manzala population at 200 mM NaCl and the lowest content was in the Wadi Asyuti population with 200 and 400 mM NaCl. In contrast, Chl b content of salt-treated plants of the three populations showed a non-significant change from their control (Figure 8). Difference in Chl a/b ratio between the three populations was also observed, the Manzala population had the highest Chl a/b ratio compared to the other populations regardless of the treatment. Interestingly, the Chl a/b ratio increased under high salinity (200 and 400 Mm NaCl) in Manzala populations while declining in Wadi Asyuti and Wadi Houf under the same concentrations (Figure 9).

**Figure 10:**
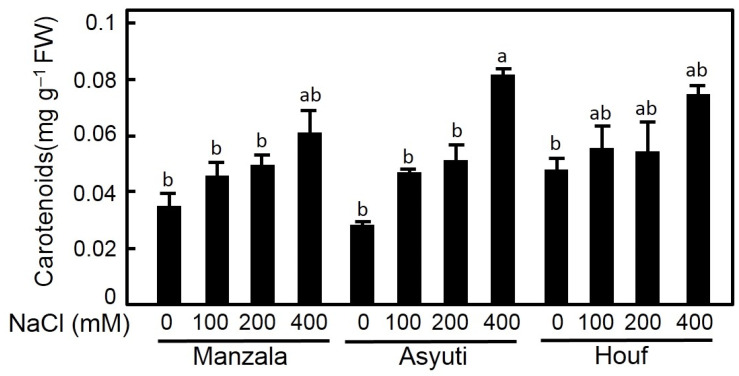
Effects of 45 days salt treatments (0, 100, 200 and 400 mM NaCl) on carotenoids in *Z. coccineum* from Manzala, Wadi Asyuti and Wadi Houf. Value represents the mean of 3 replicates ± standard error, *p*-value < 0.05. For variables denoted with the same letter, the difference is not statistically significant.

The impact of salinity on carotenoid content was significant (Figure 10). Carotenoids of three populations were promoted at all salinity levels and the Wadi Asyuti population showed higher carotenoids under 400 mM NaCl compared to other treatments.

For Manzala, our results showed significant positive correlation under salinity between germination percentage with germination index, shoot length, leaf area and Chl b. In contrast, germination percentage negatively correlated with recovery percentage, Chl a, carotenoids and Chl a/b. On the other hand, germination percentage of Wadi Asyuti and Houf had positive correlation with GRI, leaf area, shoot length, Chl a and Chl a/b while negatively correlated with recovery, Chl b and carotenoids (Table S1).

### 2.5. Isozyme Analyses

**Figure 11:**
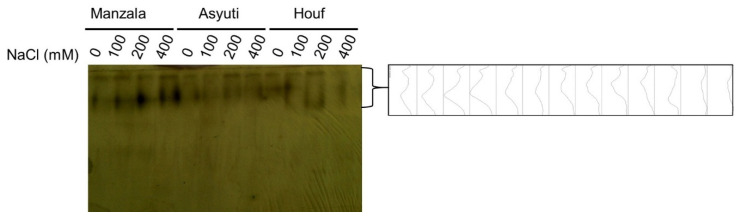
Esterase activity of *Z. coccineum* plants after 45 days incubation under 0, 100, 200 and 400 mM NaCl.

**Figure 12:**
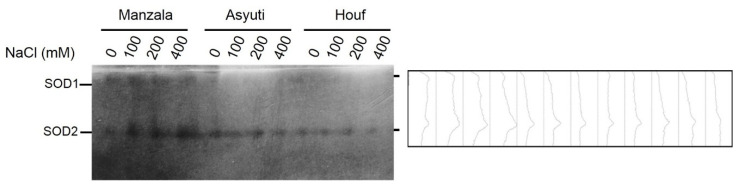
Superoxide dismutase activity of *Z. coccineum* plants after 45 days incubation under 0, 100, 200 and 400 mM NaCl.

One esterase locus and one superoxide dismutase (SOD2) loci were observed in the control and salt-treated plants of three populations while SOD1 was only detected in the Manzala population (Figure 11 and Figure 12). Whereas esterase and SOD2 activity in the Manzala population was promoted in all salinity levels, Wadi Asyuti and Wadi Houf populations had steady esterase activity in all salt-treated plants compared to their control. On the other hand, SOD1 had the same activity in the Manzala population under different conditions.

## 3. Discussion

*Zygophyllum coccineum* has a wide distribution in coastal areas, limestone Wadis and deserts in Egypt. Unpalatability of this plant and its stress tolerance play a pivotal role in its abundance. Germination success and seedling establishment are controlled by surrounding environmental conditions in addition to the ecological factors of parental generation [38]. To explore maternal environmental effects on seed germination and seedling growth of *Z. coccineum*, three populations from Manzala (coastal area), Wadi Houf and Wadi Asyuti (desert) were compared under saline conditions (100–400 mM NaCl). Our results indicate that seeds from the desert (Wadi Asyuti and Wadi Houf) had slightly higher tolerance to moderate salt stress (100–200 mM NaCl) than coastal seeds at the germination stage, especially seeds from Wadi Houf. However, germination at high salt stress (400 mM NaCl) was shown only in seeds from a coastal area (Manzala).

Similar results were observed in the facultative halophytes *Suaeda aegyptiaca* and *Anabasis setifera*; the seeds from non-saline populations had higher germination percentage compared to seeds of the saline populations under salinity [39,40]. Furthermore, Begcy et al. [41] reported that overexpression of the drought-tolerant sugarcane gene enhanced germination rate in tobacco seeds under salt stress conditions. Therefore, we assumed that the desert populations (Wadi Houf and Wadi Asyuti) express some tolerance genes that alleviate salt stress during the germination process.

On the other hand, the good germination performance of desert populations, especially Wadi Houf seeds, may be due to their heavier mass and larger length than those from Manzala. Several studies reported a correlation between seed mass and germination efficiency, for example, large seeds of *Suaeda maritima, Suaeda aegyptiaca, Anabasis setifera* and *Salicornia europaea* showed a higher rate of germination compared to small seeds [39,40,42,43]. Rapid seed germination of three populations suggests an adaptive strategy of this species to establish their seedlings during the short period of rainfall in the Egyptian desert. In addition to germination under salt stress conditions, recovery of germination from saline conditions is considered a criterion for seeds’ salt tolerance [44]. It has been reported that germination recovery enhanced with increases of salt level [15,45,46]. Our results also showed increasing recovery percentage of the three populations with increases of NaCl concentration with the highest germination recovery occurring in Manzala seeds under different salt levels. These results suggest that Manzala seeds may start their germination after dilution of salt by heavy rainfall.

Glycophytes and some halophytes have optimal growth in the absence of salt [45,47,48]. In contrast, the growth of some dicotyledonous halophytes such as *Suaeda maritima* and *Salicornia europea* was enhanced in saline conditions [49,50]. In the present study, salinity caused a significant inhibition of shoot length of the three populations. Interestingly, the lowest reduction was observed in Manzala seeds, this suggests that maternal effects contribute to salt tolerance during the seedling growth stage. To the same context, insignificant reduction in leaf area of Manzala and Wadi Asyuti under moderate salinities may be due to increases of succulence under salt stress condition. Many authors have reported the increase of succulence as an adaptive strategy of some halophytes under salinity such as *Sarcocornia natalensis* and *Halosarcia pergranulata* [51,52,53]. In contrast, the highest leaf area appeared in the Wadi Houf population in non-saline conditions which may be related to the seed size of this population which tends to produce large leaves [54].

Photosynthetic pigment content is considered a reliable biomarker for salt tolerance in plants. However, the behavior of their concentration is species dependent. For example, salt stress decreased photosynthetic pigments in *Salicornia prostrate, Suaeda prostrate, Salicornia persica* and *Salicornia europea* [50,55]. The decreasing of chlorophyll pigments under salinity could be attributed to the increase of chlorophyllase activity which leads to chlorophyll degradation [55,56,57]. In contrast, Rabhi et al. [58] reported the enhancement of pigment content in two obligate halophytes, *Tecticornia indica* and *Sesuvium portulacastrum*, under salinity compared to controls. In the present study, the highest chlorophyll a content was detected under salinity (200 mM NaCl) in the Manzala population, whereas the lowest rate was observed in Wadi Houf and Wadi Asyuti under the same conditions. These results suggest that the Manzala population is more salt tolerant than the populations from Wadi Houf and Wadi Asyuti. Similar behavior was observed in *Juncus* species where Chl a content was decreased in the more sensitive *J. articulates* under salt stress than the tolerant taxa *J. maritimus* and *J. acutus* [59]. On the contrary, the insignificant differences of Chl b in all populations under non-saline and saline conditions and the promotion of Chl a/b ratio in only the Manzala population with its decrease in Wadi Asyuti and Wadi Houf populations under high salinity suggest adaptive responses by both populations to saline and desert habitats. The halophytes usually increase the Chl a/b ratio under salinity while xerophytes are adapted to drought stress and high temperature conditions due to decreases of the Chl a/b ratio and increases of chlorophyll thermostability [58,60,61]. Furthermore, the negative correlation between germination percentage of the Manzala population with Chl a and Chl a/b and their positive correlation with the same parameters in Wadi Asyuti and Wadi Houf suggest the possibility of using these criteria in discriminating between saline and desert adapted populations.

On the other hand, the increase of carotenoid content in salt-stressed samples of the three populations suggests their role as antioxidant under salt stress conditions.

Esterases have a vital role in the developmental processes such as elongation of the stem and cellular adhesion [62]. Mohamed et al. [46] reported that esterase activity was increased in the germinated seeds of *Pancratium maritimum* under moderate salinity (50–200 mM NaCl). Likewise, the increase of esterases was observed in the leaves of halophytes *Centaurea ragusina* and Suaeda *maritima* under moderate salt stress conditions of 150–400 mM NaCl [63,64]. In the present study, increase of esterase activity under salt stress was only observed in the Manzala population suggesting its role in the salt tolerance of the Manzala population. Superoxide dismutase plays a vital role in the plant growth under environmental stress which is due to their mechanism of alleviation of oxidative damage under abiotic and biotic stress [45,65]. Mohamed et al. [20] reported the increase of SOD activity of Japanese halophyte *Suaeda maritima* under salt stress (400 mM NaCl). Furthermore, promoted SOD activity was observed in *Gypsophila oblanceolata* under 100 mM NaCl [45]. Our data showed an increase of SOD activity in the Manzala population under saline conditions (100–400 mM NaCl) compared to other populations. This result indicated the importance of SOD activity in the salt tolerance of the Manzala population.

## 4. Conclusions

*Z. coccineum* from saline and desert habitats showed different behavior during germination and seedling growth under salinity. The population from the saline habitat (Manzala) had a higher germination percentage and recovery especially under high salinity. Furthermore, Manzala seedlings showed higher performance regarding shoot height, leaf area, Chl a, Chl a/b, esterase and superoxide dismutase activities than desert populations. Therefore, maternal salinity effects clearly appeared in the Manzala population at the germination and seedling stages. The salt tolerance of Manzala seeds during recovery and seedling growth suggested an adaptive maternal salinity effect. The ability of *Z. coccineum* to adapt plays a vital role in its wide distribution because this plant has a low chance for dispersal by rain or wind through the arid conditions of Egypt, it also considered as an unpalatable food.

## 5. Materials and Methods

### 5.1. Plant Material

Mature seeds of *Zygophyllum coccineum* were collected in October 2016 on the Manzala coast (31°12′22” N, 32°1′38″ E), Wadi Asyuti (27°34′17″ N, 31°49′86″ E) and Wadi Houf (29°87′35″ N, 31°43′31″ E) in Egypt. For each population, seeds were randomly gathered and mixed together from the whole individual population (20–30 plants) to represent the genetic variation of the population. After drying for two weeks at 25 °C, the seeds were stored in paper bags with silica gel until the start of the experiments in December 2016. In Manzala, *Zygophyllum coccineum* is associated with *Zygophyllum album, Arthrocnemum macrostachyum, Halocnemum strobilaceum* and *Suaeda fruticosa*. The Wadi Asyuti population is associated with *Fagonia arabica L., Zilla spinosa (L.), Salsola imbricate,* and *Bassia muricata*. Additionally, in Wadi Houf, the associated species are *Salsola imbricate* subsp. *imbricata, Schouwia purpurea* and *Zilla spinosa.* The mean annual temperature for the last five years for Manzala, Wadi Asyuti and Wadi Houf were 22 °C, 24 °C and 24.2 °C (Table 2); the mean annual precipitation is 75.9 mm, 3.8 mm and 38.7 mm (https://www.worldweatheronline.com accessed 23 August 2020); and the total dissolved salts were 43,800, 4300 and 595 ppm, respectively [66,67,68].

### 5.2. Effects of Salinity on Germination and Recovery

To investigate seed germination of *Z. coccineum* under saline conditions, three replicates of 20 seeds were germinated in dishes on filter paper (Whatman No. 1) wetted with 5 mL of distilled water, 100, 200 and 400 mM NaCl at 23 °C in the dark. Germination percentage was recorded daily for 15 days. A seed was recorded as germinated when radicle length was 1–2 mm. Germination rate was calculated according to the following equation: Germination velocity = ΣG/t, where G is the percentage of germinated seeds at 2-day intervals and t is the total period of germination [69].

To determine seed recovery percentage, the salt treated un-germinated seeds were transferred to distilled water and germination was counted daily for 15 days. Recovery percentage was calculated by the equation: recovery percentage = (A − B/C) × 100, where A is total number of seeds germinated after being transferred to distilled water, B is seeds germinated in saline solution and C is the total number of seeds [70].

### 5.3. Effects of Salinity on Plant Growth

The seeds were germinated in pots filled with a mixture of sand, peat moss and vermiculite soil (1:1:1). Germinates were grown for 45 days at 23 °C under 300 µmol m^−2^ s^−1^ (16 h light/ 8 h dark) and irrigated every third day with 20% MS solution. For the stress treatment, 45-day-old plants were irrigated with 20% MS solution containing 100, 200 or 400 mM NaCl for 45 days and then the plants were harvested and used for the following analyses.

#### 5.3.1. Growth Parameters

Leaf area was measured using the graph paper method, leaves were traced on graph paper then area was calculated by counting of squares [71]. Shoot length was measured with a ruler [72].

#### 5.3.2. Photosynthetic Pigments Measurement

Chlorophylls and carotenoids were extracted by acetone from 100 mg of fresh leaf tissue. The mixture was subjected to centrifugation for 10 min. The supernatant absorbance at 663, 645 and 452 nm was used for the analyses of pigments using spectrophotometer (Jenway, UK). Chl a, Chl b and carotenoids were calculated according to [73].

#### 5.3.3. Esterase and SOD Activity

Half a gram of leaves was ground in 1 mL of 20 mM Tris HCl buffer, pH 7.8, containing 1 mM dithiothreitol, 1 mM EDTA, and 20 mg polyvinyl-polypyrrolidone. The homogenized samples were centrifuged for 10 min at 14,000× *g* and 4 °C. The supernatant was collected and then was used for the detection of esterase and SOD activities. Protein was calculated according to the method proposed by Bradford [74] and with BSA as standard. Equal amounts of protein samples were loaded to non-denaturing polyacrylamide gel prepared according to the Laemmli system without SDS [75]. The running condition was 100 V for 120 min at 4 °C.

For SOD staining, gels were soaked for 30 min in 200 mM potassium phosphate buffer of pH 7.8, containing EDTA (1 mM), nitro blue tetrazolium (0.24 mM) and riboflavin (0.1 mM). Isozymes were visualized as colorless bands under fluorescence light. The negative band is presented as positive for clarification [26].

Esterases were detected by pre-soaking the gel for 30 min in 100 mL of 0.1 M sodium phosphate buffer (pH 7) containing 0.1 g fast blue RR and 15 mg *α*-naphthyl acetate (dissolved in 0.5 mL acetone) at 37 °C in the dark. The gel was then transferred and stored in 7% acetic acid [76]. The bands in both gels were transformed into density profiles using Image J software [77].

### 5.4. Statistical Analysis

All parameters were expressed as means with standard error and Levene’s test was applied to examine the homogeneity of variances of all data. Final germination percentage (percentage at 15 days), recovery and germination rate index (GRI) were subjected to Kruskal–Wallis H-test followed by a Mann–Whitney post-hoc test. Other parameters were subjected to one-way analysis of variance (ANOVA) and Tukey test. Two-way ANOVA was applied to determine the impact of maternal habitat, salinity and their interaction with all parameters. All statistical analyses were performed using SPSS 16.0 statistical software. Means comparison was set at *p* < 0.05 and values denoted by the same letter are not significantly different.

## Figures and Tables

**Table 1:** Two-way ANOVA analysis showing the mean squares, F and *p*-values of the effects of salinity, maternal habitat and their interaction on all studied parameters.

Growth Parameters	Salinity	Habitat	Salinity × Habitat
	Degree of Freedom	Degree of Freedom	Degree of Freedom
	Mean Squares	Mean Squares	Mean Squares
	F-Value	F-Value	F-Value
	*p*-Value	*P*-Value	*p*-Value
Seed germination	3	2	6
	618.4	1.79	15.25
	152.8	0.222	0.628
	<0.001	<0.001	ns
Recovery	2	2	4
	6.4	81.7	0.444
	24.5	313.5	1.70
	<0.001	<0.001	ns
Shoot length	3	2	6
	56.4	48.6	2.1
	134.17	115.5	5.10
	<0.001	<0.001	<0.001
Leaf area	3	2	6
	0.102	0.015	0.013
	106.59	16.17	14.0
	<0.001	<0.001	<0.001
Chl a	3	2	6
	0.001	0.023	0.001
	1.61	27.73	1.06
	ns	<0.001	ns
Chl b	3	2	6
	0	0.003	0.001
	0.395	3.81	0.689
	ns	<0.001	ns
Carotenoids	3	2	6
	0.002	0	0
	19.45	3.31	1.36
	<0.001	ns	ns
Chl a/b	3	2	6
	0.022	2.909	0.378
	0.065	8.81	1.14
	ns	0.01	ns
Germination rate index	3	2	4
	2337.16	2.905	13.74
	385.7	0.645	2.26
	<0.001	ns	ns

**Table 2:** The mean annual weather conditions of last five years of three habitats.

Habitat	Air Temperature (°C)	RH (%)	Rainfall (mm)	Total Dissolved Salts
Manzala	22	62.2	75.9	43,800 [29]
Wadi Houf	24.2	43.9	38.7	4300 [30]
Wadi Asyuti	24	31.5	3.8	595 [31]

## Supplementary Materials

The following are available online at https://www.mdpi.com/2223-7747/9/11/1504/s1, Table S1: Correlation coefficient between germination, seedling growth biochemical parameter in *Z. coccineum* from Manzala, Wadi Asyuti and Wadi Houf under 34 salinity conditions.
