# Supplementary material for: Influence of Maternal Habitat on Salinity Tolerance of Zygophyllum coccineum with Regard to Seed Germination and Growth Parameters
Source: Plants (Basel). 2020 Nov 6;9(11):1504. doi: 10.3390/plants9111504 (PMC7694771; doi:10.3390/plants9111504)
Supplement: Supplementary file 1 [file plants-09-01504-s001.pdf]

plants

Article

Influence of Maternal Habitat On Salt Tolerance During Germination and Growth in *Zygophyllum coccineum*

Elsayed Mohamed [1,*], Ahmed M. M. A. Kasem [1], Adil A. Gobouri [2], Amr Elkelish [3] and Ehab Azab [4,5]

1 Botany & Microbiology Department, Faculty of Science (Assiut), Al-Azhar University, Assiut 71524, Egypt; amkasem@azhar.edu.eg
2 Department of Chemistry, College of Science, Taif University, P.O. Box 11099, Taif 21944, Saudi Arabia; a.gobouri@tu.edu.sa
3 Botany Department, Faculty of Science, Suez Canal University Ismailia, Ismailia 41522, Egypt; amr.elkelish@science.suez.edu.eg
4 Department of Biotechnology, College of Science, Taif University, P.O. Box 11099, Taif 21944, Saudi Arabia; e.azab@tu.edu.sa
5 Botany and Microbiology Department, Faculty of Science, Zagazig University, Zagazig, 44519 - Sharkia-Egypt

* Correspondence: sayedmohamed@azhar.edu.eg; Tel.: (+20-100-854-7019)

Table S1. Correlation coefficient between germination, seedling growth biochemical parameter in *Z. coccineum* from Manzala, Wadi Asyuti and Wadi Houf under salinity condition.

Location	Parameters	Germination	Index	Recovery	Length	Area	Chla	Chlb	Carotenoids	Chla/Chlb
Manzal	Germination	1	**0.997****	-0.990**	**0.853****	**0.690***	-0.051-	0.117	-0.673*	-0.347
	Index	**0.997****	1	-0.982**	**0.871****	**0.707***	-0.037-	0.102	-0.679*	-0.327
	Recovery	-0.990**	-0.982**	1	-0.829**	-0.705*	-0.003-	-0.140	**0.669***	0.351
	Length	**0.853****	**0.871****	-0.829**	1	**0.829****	0.153	0.219	-0.834**	-0.351
	Area	**0.690***	**0.707***	-0.705*	**0.829****	1	0.355	0.474	-0.860**	-0.547
	Chla	-0.051-	-0.037-	-0.003-	0.153	0.355	1	0.279	-0.075	0.039
	Chlb	0.117	0.102	-0.140-	0.219	0.474	0.279	1	-0.513	-0.901**
	Carotenoids	-0.673*	-0.679*	**0.669***	-0.834**	-0.860**	-0.075-	-0.513	1	**0.653***
	Chla/Chlb	-0.347-	-0.327-	0.351	-0.351-	-0.547-	0.039	-0.901**	**0.653***	1
Wadi Assiuty	Germination	1	**0.992****	-0.992**	**0.968****	**0.838****	0.448	-0.091	-0.842**	0.092
	Index	**0.992****	1	-0.980**	**0.968****	**0.810****	0.380	-0.071	-0.816**	0.019
	Recovery	-0.992**	-0.980**	1	-0.962**	-0.816**	-0.451	0.101	**0.818****	-0.117
	Length	**0.968****	**0.968****	-0.962**	1	**0.857****	0.494	-0.128	-0.814**	0.137
	Area	**0.838****	**0.810****	-0.816**	**0.857****	1	**0.521**	-0.256	-0.935**	0.308
	Chla	0.448	0.380	-0.451-	0.494	0.521	1	-.188-	-0.411	**0.508**
	Chlb	-0.091-	-0.071-	0.101	-0.128-	-0.256-	-0.188-	1	0.031	**0.822****
	Carotenoids	-0.842**	-0.816**	**0.818****	-0.814**	-0.935**	-0.411	.031	1	-0.126-
	Chla/Chlb	**0.092**	0.019	-0.117	0.137	0.308	**0.508**	-0.822**	-0.126-	1
Wadi Houf	Germination	1	**0.987****	-0.979**	**0.986****	**0.934****	0.490	-0.071-	-0.560-	**0.586***
	Index	**0.987****	1	-0.965**	**0.968****	**0.957****	**0.530**	-0.067-	-0.496-	**0.633***
	Recovery	-0.979**	-0.965**	1	-0.961**	-0.891**	-0.442-	0.165	0.476	-0.572-
	Length	**0.986****	**0.968****	-0.961**	1	**0.900****	0.471	-0.132-	-0.536-	**0.610***
	Area	**0.934****	**0.957****	-0.891**	**0.900****	1	**0.577***	-0.023-	-.0522-	**0.670***
	Chla	0.490	**0.530**	-0.442-	0.471	**0.577***	1	0.479	0.194	**0.846****
	Chlb	-0.071-	-0.067-	0.165	-0.132-	-0.023-	0.479	1	0.007	-0.049-
	Carotenoids	-0.560-	-0.496-	0.476	-0.536-	-0.522-	0.194	0.007	1	0.198
	Chla/Chlb	**0.586***	**0.633***	-0.572-	**0.610***	**0.670***	**0.846****	-0.049-	0.198	1

** Correlation is significant at the 0.01 level (2-tailed); * Correlation is significant at the 0.05 level (2-tailed).
